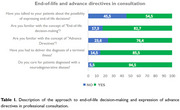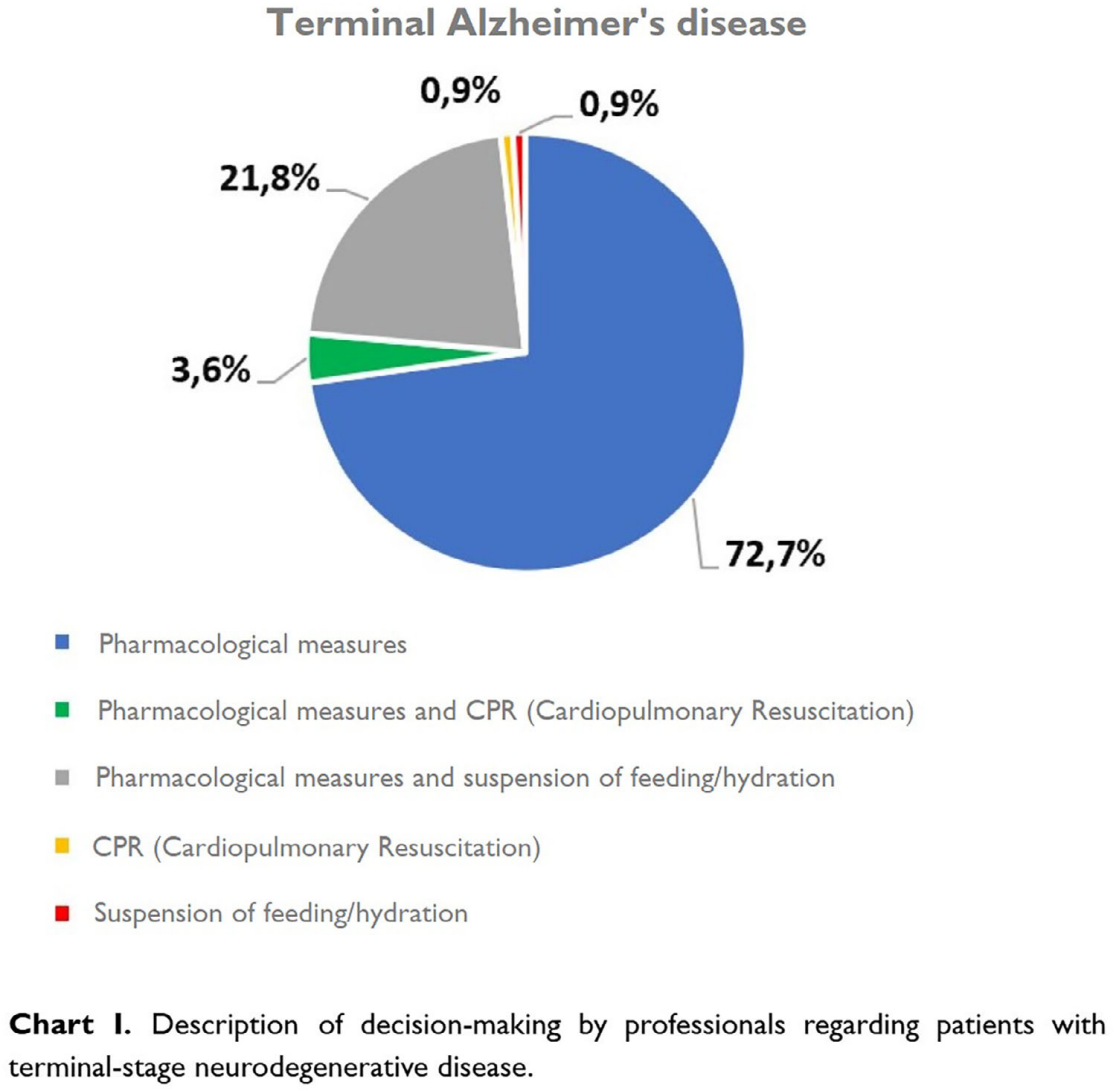# Perception of bioethical‐legal issues among professionals assisting patients with neurodegenerative diseases

**DOI:** 10.1002/alz70858_104642

**Published:** 2025-12-26

**Authors:** Ximena Giordano, Marcelo Rugiero, Rosa Angelina Pace, Maria Cecilia Fernández

**Affiliations:** ^1^ Hospital Italiano de Buenos Aires, Buenos Aires, Argentina; ^2^ Hospital Italiano de Buenos Aires, Buenos aires, Argentina; ^3^ Hospital Italiano de Buenos Aires, CABA, Argentina

## Abstract

**Introduction:**

Knowledge about bioethics in medicine and the application of ethical principles when addressing patients with neurodegenerative diseases has evolved in recent years. To ensure quality care, it is necessary for professionals to receive proper training in bioethical issues. Although bioethics is incorporated into the curricula of health‐related degrees, there is an educational gap in legal aspects and some hesitation when offering end‐of‐life measures. The aim of this study was to evaluate the perception of bioethical‐legal issues among professionals who assist patients with neurodegenerative diseases.

**Methods:**

A descriptive cross‐sectional study was conducted; data were collected through an anonymous self‐administered survey via an online form regarding bioethical, legal, and personal definitions related to knowledge of the subject. The survey included professionals from the specialties of neurology, psychology, neuropsychology, psychiatry, geriatrics, and related fields. Average and dichotomous values were calculated to obtain the results.

**Results:**

Data were collected from 110 participants with an average of 16.2 years of professional experience. Medical profession accounted 86.4%; neurology specialty represented 93.7%. The majority were in the field of cognition. 94.5% were familiar with the concept of bioethics and worked with patients with neurodegenerative diseases; 76.4% had a clear understanding of advance directives, although only 3.6% emphasized the importance of patient autonomy. Within the legal framework, 96.4% considered appropriate to respect patient autonomy and their right to express advance directives. Most participants opted to administer pharmacological measures for comfort for terminally ill patients. In the context of their own experience with a neurodegenerative disease, 40.9% of professionals indicated they would request palliative sedation and a “do not resuscitate” order. Finally, 97.3% of professionals expressed a desire to know their diagnosis if they were to suffer from a neurodegenerative disease.

**Discussion:**

Many professionals dealing with neurodegenerative diseases encounter situations where patients and families question disease progression and time for life planning. Most of them appear to have an adequate understanding of bioethical and legal principles. However, an educational gap is evident regarding the expression of the patient's will. We consider these findings highlight areas for improvement in providing comprehensive care and can help guide future learning in these complex scenarios.